# Effect of Root and Mycelia on Fine Root Decomposition and Release of Carbon and Nitrogen Under *Artemisia halodendron* in a Semi-arid Sandy Grassland in China

**DOI:** 10.3389/fpls.2021.698054

**Published:** 2021-09-01

**Authors:** Xinping Liu, Yongqing Luo, Li Cheng, Hongjiao Hu, Youhan Wang, Zhong Du

**Affiliations:** ^1^Naiman Desertification Research Station, Northwest Institute of Eco-Environment and Resources, Chinese Academy of Sciences, Lanzhou, China; ^2^School of Geographical Sciences, China West Normal University, Nanchong, China; ^3^Forest Dynamics, Swiss Federal Institute for Forest, Snow and Landscape Research WSL, Birmensdorf, Switzerland

**Keywords:** artemisia halodendron, mycelia, fine root, root decomposition, ingrowth core method

## Abstract

Plant fine root turnover is a continuous process both spatially and temporally, and fine root decomposition is affected by many biotic and abiotic factors. However, the effect of the living roots and the associated mycorrhizal fungal mycelia on fine root decomposition remains unclear. The objective of this study is to explore the influence of these biotic factors on fine root decomposition in a semi-arid ecosystem. In this study, we investigated the effect of fine roots and mycelia on fine root decomposition of a pioneer shrub (*Artemisia halodendron*) in Horqin sandy land, northeast China, by the ingrowth core method combined with the litterbag method. Litterbags were installed in cores. Results showed that core a allowed the growth of both fine roots and mycelia (treatment R + M), core b only allowed the growth of mycelia (treatment M), and in core c the fine root and mycelia growth were restricted and only bulk soil was present (treatment S). These findings suggest that the process of root decomposition was significantly affected by the living roots and mycelia, and carbon (C) and nitrogen (N) concentration dynamics during root decomposition differed among treatments. Mycelia significantly stimulated the mass loss and C and N release during root decomposition. Treatment R + M significantly stimulated the accumulation of soil total C, total N, and organic N under litterbags. The mycelia significantly stimulated the accumulation of the inorganic N (ammonium-N and nitrate-N) but the presence of fine roots weakened nitrate-N accumulation. The presence of living roots and associated mycelia strongly affected the process of root decomposition and matter release in the litter-soil system. The results of this study should strengthen the understanding of root-soil interactions.

## Introduction

Litter decomposition is a major process within nutrient cycling and energy flows in terrestrial ecosystems. Fine root turnover is an important source of soil carbon (C) and nitrogen (N) during the development of plant roots (Jackson et al., 1997; Silver and Miya, 2001). The carbon contribution of fine roots to terrestrial ecosystems is of great importance because of their rapid turnover despite the relatively small proportion of fine roots (Silver and Miya, 2001; Stover et al., 2010; Finér et al., 2011; Huang et al., 2012; Sariyildiz, 2015). The study of Jackson et al. (1997) showed that plants in terrestrial ecosystems store 38.1 × 10^9^ mg of C in their fine roots, which is ~5% of the size of the atmospheric C pool. Moreover, studies have also shown that soil C accumulates even more through plant root decomposition than through the aboveground biomass (Usman et al., 2000; Kätterer et al., 2011; Bolinder et al., 2012). Therefore, root decomposition is the main and stable source for the accumulation of soil matter such as organic C and nutrient elements (Luo et al., 2016a; Liebmann et al., 2020).

The production of root exudates is an important process by which plants influence the material cycle of the plant-soil system through roots (Phillips et al., 2011; Zhang et al., 2015; Zwetsloot et al., 2018). However, the precise influence of root exudates on soil C and N cycle is still uncertain. Taking soil C as an example, root exudates can promote the decomposition of soil organic carbon (SOC) through the improvement of rhizosphere soil microbial activity and soil enzyme activity (Rukshana et al., 2012; Girkina et al., 2018). Conversely, it has also been shown that the root exudates can limit the decomposition of SOC by inhibiting soil microbial activity and enzyme activity in the rhizosphere, therefore promoting the accumulation of SOC (Zhang et al., 2015; Zwetsloot et al., 2018). Furthermore, the current studies have mainly focused on the forest ecosystem, while the related study in semi-arid degraded grassland remains scarce.

Most existing studies about litter decomposition have reported that root exudate can stimulate litter decomposition through promoting population and activity of soil microorganisms (Baudoin et al., 2003; Landi et al., 2006; Técher et al., 2011) and altering the form of soil N (Nardi et al., 2002; Landi et al., 2006). However, in the case of water-limited arid or semi-arid zones, the absorption of water by plant roots leads to the decrease of soil moisture content (Schwinning and Ehleringer, 2001; Loik et al., 2004; Zhou et al., 2015), which limits litter decomposition. Therefore, it remains uncertain whether the presence of plant roots would stimulate litter decomposition *via* its promotion effect derived from root exudates or inhibit litter decomposition because of the reduced soil moisture content.

Mycorrhiza plays important role in plant root growth and soil C and N cycle in the plant-soil system (Cheng et al., 2012; Phillips et al., 2012). The colonization of arbuscular mycorrhizal fungi (AMF) is considered to promote the decomposition of the aboveground litter (Schädler et al., 2010), while it showed insignificant influence on root litter (Urcelay et al., 2011). N is considered a key factor in the process of mycorrhiza influence on litter decomposition. The AMF could enhance the decomposition rate of litter and obtain inorganic N released from the litter during its decomposition, and thus mycelium growth was promoted by effective utilization of decomposition products (Hodge et al., 2001). The study of Cheng et al. (2012) showed that AMF accelerated litter decomposition by changing the contents of ammonium-N and nitrate-N in the soil. Most of the relevant studies at present have focused on forest ecosystems with non-limited water conditions, and the research objects are mainly aboveground litter. In water-limited ecosystems with less precipitation, the mechanism of the mycorrhizal fungal mycelia effect on root decomposition remains unclear.

The previous studies in Horqin degraded sandy grassland (a typical semi-arid climate region in northeast China) found that soil C under litterbags varied greatly after 1 year of decomposition for the fine roots of *Artemisia halodendron* compared with no litterbags under natural conditions (Luo et al., 2016a). This is a typical AMF infestation species (Te, 2007) and is one of the dominant shrubs in Horqin sandy land and plays an important role during the process of dune stabilization (Huang et al., 2012; Luo et al., 2020a). Meanwhile, the decomposition rate of the fine root of *A. halodendron* under natural conditions also differed significantly compared with another study in the same region (Li et al., 2016; Luo et al., 2020b). Therefore, we preliminarily hypothesized that the difference of water and temperature in the initial decomposition stage (caused by the difference in the starting date) would be the main factor for these differences. Thus, we carried out an experiment focusing on the effect of starting time on root decomposition of *A. halodendron* (Luo et al., 2020b). It was found that the fine root decomposition rate of *A. halodendron* at different starting times differed significantly; however, the difference was small, and the root decomposition is likely to be affected by other factors such as the variance of soil moisture and its interaction with soil temperature (Luo et al., 2016a, 2020b). Given that the plant community in this area is dominated by short-lived annual species, and the composition and structure of plant community varied greatly in both spatial and temporal scales (Wang, 1989; Zuo et al., 2009; Duan et al., 2014; Wang et al., 2018), we then hypothesized that the existence of the living roots and their related processes such as mycorrhizal fungal mycelia and root exudates in the vicinity of litterbags might affect the process of root decomposition and element transformations in the litter-soil system. Therefore, a litter decomposition experiment under the canopy of *A. halodendron* was conducted by the ingrowth core method combined with the litterbag method in a semi-fixed dune in the Horqin sandy land. The specific objectives of this study were as follows: (1) to clearly describe the effects of plant fine roots and related mycorrhizal fungal mycelia on the decomposition of fine root (0–2 mm) of *A. halodendron* under the canopy, and (2) to clarify the effects of fine roots and mycelia of *A. halodendron* on soil C and N variation during fine root decomposition in semi-arid degraded sandy grassland. Overall, the study aimed to strengthen the understanding of the influence of living plant roots and mycelia on fine root decomposition and soil C and N variations under water-limited conditions.

## Materials and Methods

### Study Site

The study was conducted at the Naiman Desertification Research Station of the Chinese Academy of Sciences (42°58′ N, 120°43′ E; elevation 360 m.a.s.l.), in the southwestern part of the Horqin sandy land, located in the eastern part of Inner Mongolia Autonomous Region in China. This region belongs to the cold temperate zone, with a semi-arid continental monsoon climate. Mean annual precipitation and annual mean potential evaporation are 343 and 1,935 mm, respectively. The mean annual temperature is 6.7°C, with a minimum monthly mean temperature of −12.6°C in January and a maximum of 24.3°C in July. The soil is classified as Cambic Arenosols of sandy origin in the Food and Agriculture Organization (FAO) soil classification system (FAO, 2006). The soil is sandy, with a coarse texture (medium to coarse sand) and a loose structure, and particularly susceptible to wind erosion (Luo et al., 2020a). In semi-fixed dune, the SOC concentration ranging from 0.35 to 0.87 g m^2^ and the soil bulk density ranging from 1.57 to 1.6 g cm^3^ among soil depth of 0–100 cm (Luo et al., 2015). The landscape is characterized by sand dunes formed by grassland degradation at different stages, including mobile, semi-fixed, and fixed dunes. The state of these sand dunes can convert from each other through the influence of biotic and abiotic factors. For example, intensive human activities (especially overgrazing) have accelerated the process of desertification and large areas of fixed dunes have been converted into mobile dunes in the last century (Wang, 1989). In recent decades, the area of severely degraded mobile dune land has declined substantially because of the efficient implementation of a series of governmental protection programs (Duan et al., 2014). In the semi-fixed dune, the vegetation coverage is ranging from 30 to 60%, and the plant community is dominated by perennial shrubs, i.e., *A. halodendron, Caragana microphylla*, and *Salix gordejevii*, and some annual herbs, i.e., *Chenopodium acuminatum, Corispermum macrocarpum, Bassia dasyphylla, Artemisia scoparia*, and *Setaria viridis*.

### Experimental Design

*Artemisia halodendron* has the property of asexual reproduction, and this characteristic combined with the frequent sand burial makes it difficult to define the specific age of the plant. Age is considered an important controlling factor on plant fine root turnover and mycorrhiza colonization. This is because, with increasing age, the fine root production (Fogel, 1983) and mycorrhizal infection rate both decrease (Cheng et al., 2005). Thus, we designed a field transplantation experiment to reduce the error from plant age by specifying all the transplanted plants as about 1 year old. In this transplantation field, six plots (each 9 × 9 m) were selected, and 49 plants were planted in each plot with a 1 m spacing. Each of the plots was separated from the adjacent plots by a 1 m buffer zone. This transplanted experimental field represents the semi-fixed dunes in this region. Detailed information about this transplanted field is given in the earlier research (Luo et al., 2020a). After 4 years of growth, in April 25, 2016, *A. halodendron* with similar form were randomly chosen from these six plots with 294 plants (49 × 6) for this decomposition experiment (5 samplings × 5 replications).

At the end of October 2015, we collected root samples of *A. halodendron* by excavating to a depth of 30 cm (samples to this depth contain the majority of the roots) from semi-fixed dunes within 10 km of the station. All samples were washed carefully with tap water to remove the soil, and the fine root with a diameter of <2 mm was separated by hand. Afterward, the fine root sample was oven-dried at 65°C for 48 h. The dried root sample was cut into lengths of 2–3 cm, and 5 g portions of the roots were placed in separate nylon mesh bags (10 × 10 cm, with a 0.1-mm mesh spacing). The bags were then sealed and stored in a vacuum desiccator in the dark to prevent decomposition before the start of the incubation. We prepared a total of 80 mesh bags for use in this study (3 treatments × 5 decomposition periods × 5 duplication, and the remaining 5 bags were used for initial chemical determination).

**Figure 1:**
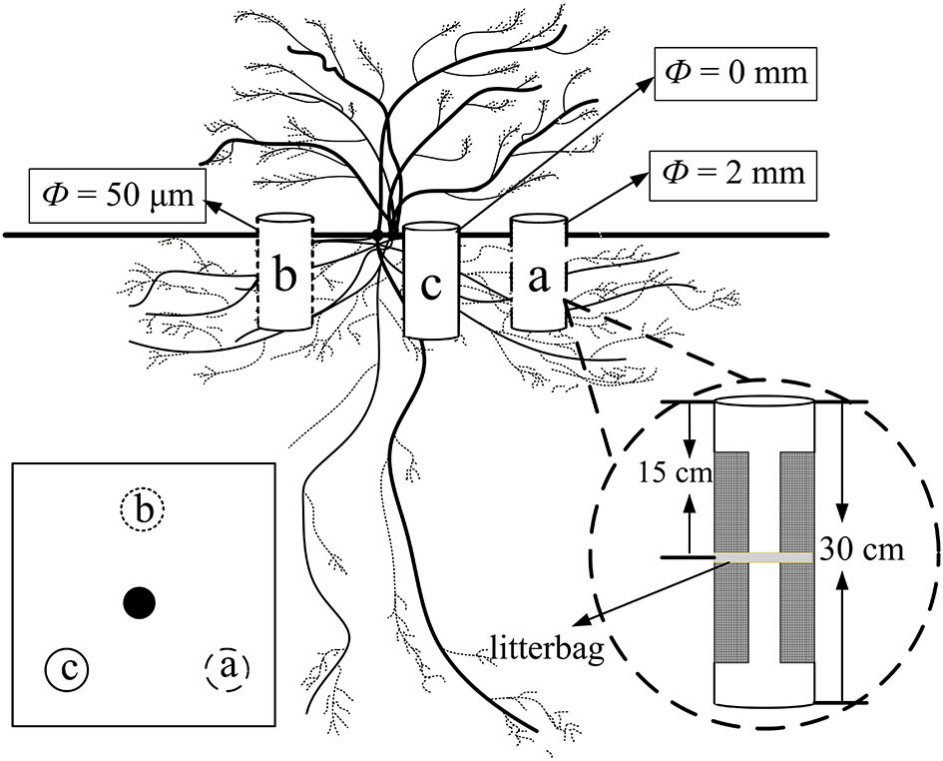
Illustration of PVC ingrowth core installation for the fine root decomposition of *Artemisia halodendron* under the canopy of an *A. halodendron* plant. Cores a and b had two windows on each side of the PVC pipe and all faced vertically toward the center of the canopy. There were no windows for core c. The mesh sizes of the nylon mesh for core a and core b were 2 mm and 50 μm, respectively; thus, core a allowed the growth of both fine root and mycelia (R + M), core b only allowed the growth of the mycelia (M), and core c contained only bulk soil (S). This group of ingrowth cores was installed at the point of half canopy width toward the center at an angle of 120°.

Three points were selected at the point of half canopy width toward the center at an angle of 120° of each plant and marked as a, b, and c, respectively (Figure 1). The ingrowth core method was modified from that used by the study of Phillips et al. (2012). One ingrowth core comprising a PVC pipe (10 cm in diameter and 30 cm in height) was installed in each plot vertically (Figure 1). Large segments (20 cm in height and 5 cm in width) of each PVC pipe at each side were cut to create “windows” to allow the growth of the fine root and mycelia for core *a* and core *b*, while there were no windows for core *c*. Windows were tightly wrapped with a mesh of two different pore sizes. The mesh size was 2 mm and 50 μm for cores *a* and *b*, respectively. Thus, core *a* allowed the growth of both fine root and mycelia (treatment R + M), core *b* only allowed the growth of the mycelia (treatment M), and both the fine root and the mycelia were restricted in core *c* and only bulk soil was present (treatment S). The windows of each core faced vertically toward the center of the canopy. The windows were 5 cm apart from the top and the bottom edge of the PVC pipe, respectively.

At the beginning of the growing season, May 4, 2016, all ingrowth cores were installed and the litterbag with 5 g fine root sample of *A. halodendron* was placed horizontally in the middle layer of each core (15 cm to the top edge of the pipe, in the middle of the window, Figure 1). All excavated soil from the ingrowth core installation was filled back to the core immediately after 2-mm sieving by the depth of every 10 cm. Thus, organic matters, including living roots and the necromass, were removed and soil bulk density was close to that in the natural state. From the beginning of the decomposition experiment, we checked the core every 5 or 7 days and fallen litter at the soil surface or germinated seedlings were all removed to reduce the influence of new plant roots and litter on the experiment.

### Sampling and Measurement

The decomposed fine root samples of each ingrowth core were collected on June 3, August 3, and October 3, 2016; May 3, 2017; October 3, 2018. On each sampling date, five litterbags for each treatment were sampled. These bags were then air-dried and stored in the fridge at 5°C in darkness until the end of the experiment. After the final sampling on October 3, 2018, all roots remaining in the litterbag, including the previously collected and stored litterbags, were washed carefully with water and then dried for 48 h at 65°C. Masses of the root samples were measured, and then they were ground to pass through a 2 mm mesh and stored in a fridge at 5°C until analysis. Then, C and N concentrations of all decomposed root samples and the control were measured using the dry combustion method with a Vario Macro Cube elemental analyzer (Elementar, Hanau, Germany).

After 1 year of decomposition, together with the fourth litter sample collection on May 3, 2017, the soil samples under the litterbag of each treatment were collected at the depths of 0–1, 1–2, 2–3, 3–4, 4–5, 5–6, and 6–10 cm with a 10 × 10 cm horizontal area directly below each litterbag (the previous studies have shown that after 1 year of root decomposition of *A. halodendron*, the potential depth of influence on soil C and N content is 0–6 cm under litter bags (Luo et al., 2016a). The collected soil was divided into two parts. The first part was preserved as a fresh sample for the determination of soil water content through the drying method (105°C for 48 h) and of nitrate-N and ammonium-N by the colorimetric method after KCl extraction. The second part was air-dried for the determination of total C and N using the dry combustion method with the Vario Macro Cube elemental analyzer.

The climate data were obtained from the meteorological station at the Naiman Desertification Research Station (http://nmd.cern.ac.cn/meta/metaData), which was located <100 m from the transplanting field.

### Statistical Analysis

The statistical analysis was conducted using version 20 of the SPSS software (www.ibm.com/software/analytics/spss/). For the fine root decomposition characterization of *A. halodendron*, we analyzed differences in the remaining mass, in the C and N concentrations, in the C:N ratio, and in the C and N remaining among the treatments (T) and decomposition time (D*t*) by using a two-way ANOVA with T and D*t* as factors. We performed multiple comparisons using the LSD test whenever the ANOVA indicated a significant difference (*P* < 0.05). The effect size of fine root and mycorrhiza on root C and N remaining was calculated by the following equations:

(1) $$\begin{array}{l} \text{Effectsize}\left(\%\right)=\left({\text{T}}_{a/b}-{\text{T}}_{c}\right)/{\text{T}}_{c}\times 100\% \end{array}$$

where T_*a*/*b*_ is the average C or N remaining in core *a* or core *b*, and T_*c*_ is the C or N remaining in core *c*.

For the soil properties under fine root litterbag of *A. halodendron*, we analyzed differences in soil moisture, total C concentration, total N concentration, C:N ratio, organic-N, inorganic-N, nitrate-N, and ammonium-N among the treatments (T), and soil depth (D) by two-way ANOVA, with T and D as factors. We performed multiple comparisons using the LSD test whenever the ANOVA indicated a significant difference (*P* < 0.05). The inorganic N concentration in this study was defined as the sum of nitrate-N and ammonium-N, and the organic N was defined as the total N minus inorganic N.

## Results

### Climate Conditions

**Figure 2:**
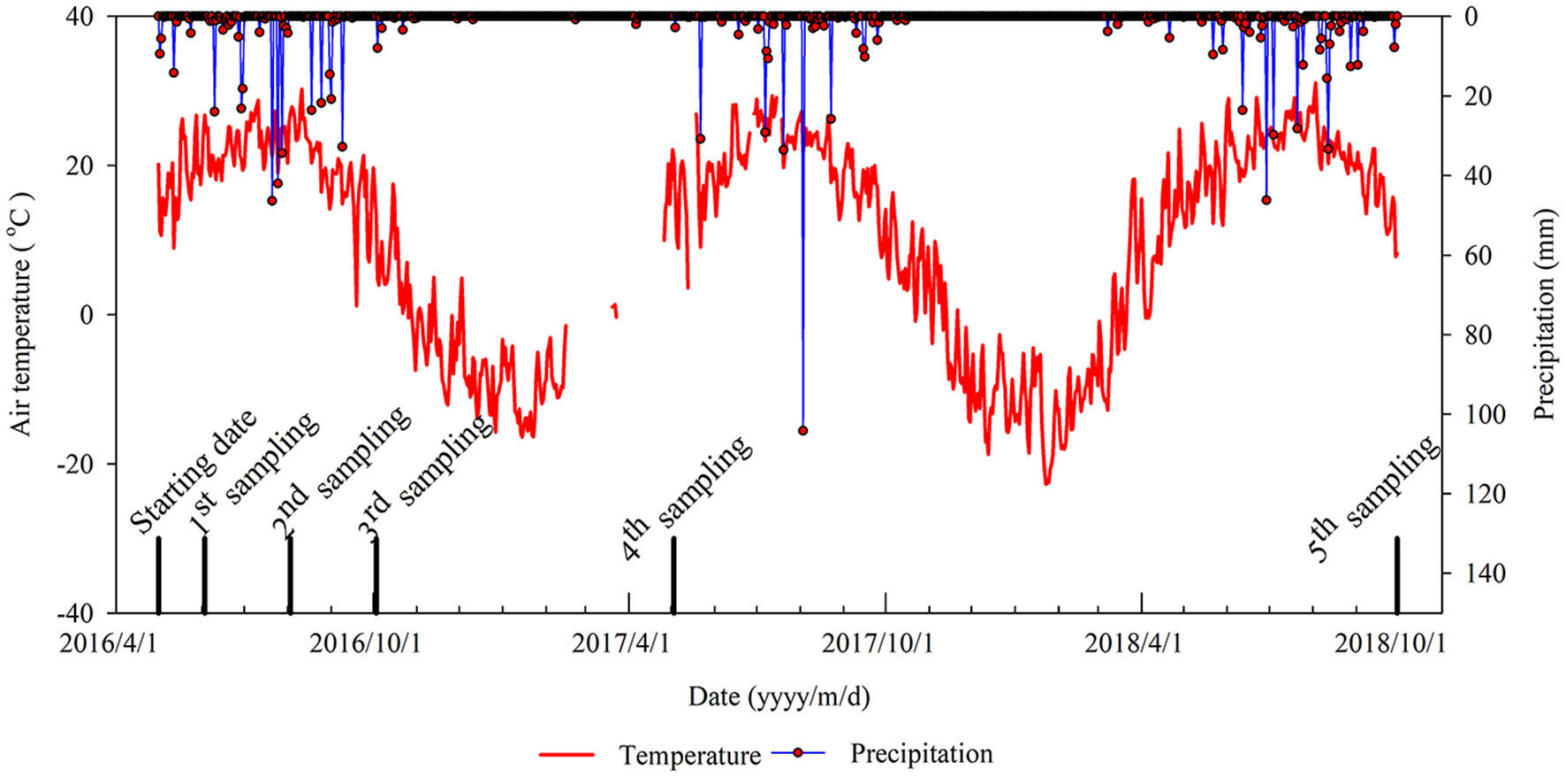
Precipitation and air temperature during the study period.

From May 4, 2016, to October 3, 2018, there were 154 precipitation events in total with a total amount of 1,009.4 mm, and there were 70 effective precipitation (daily precipitation amount > 2 mm) events with a total amount of 956.4 mm. There were 13 heavy precipitation (daily precipitation amount > 25 mm) events and the greatest amount of precipitation in 1 day was 104.2 mm on August 3, 2017. The daily mean air temperature during the experiment was 10°C, and in the growing season (May 1–October 31) daily mean air temperature ranged from 18 to 18.2°C (Figure 2).

### Fine Root Decomposition and Dynamics of C and N

**Figure 3:**
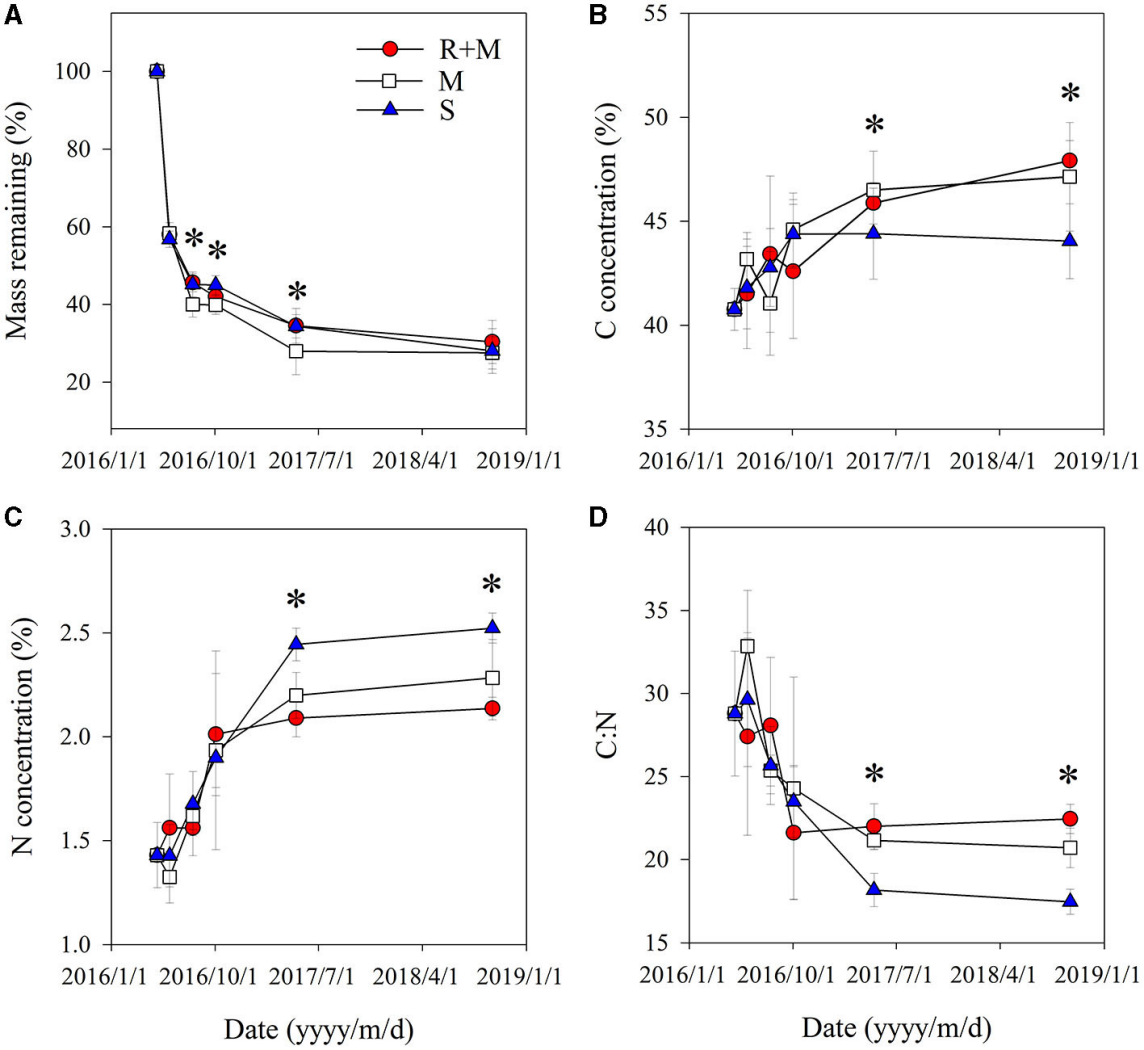
Fine root traits of *A. halodendron* during decomposition under the following three treatments: fine root and mycelia (R + M), mycelia (M), and bulk soil (S). **(A)** indicates the dynamic of mass remaining to the initial value; **(B)** and **(C)** indicate the changes of carbon (C) and nitrogen (N) concentrations, respectively; and **(D**) indicates the change of root C:N ratio. Values represent *M* ± S*D*. Values of a parameter on a given date labeled with * differed significantly among treatments (*P* < 0.05).

The fine root mass loss and concentrations of C and N of *A. halodendron* varied among decomposition times (Table 1). The mass decomposed rapidly in the initial period, in which more than 40% of the mass was lost within the first 33 days (Figure 3A). The carbon and nitrogen concentrations both increased over time, and the significant differences among treatments only existed at the latal stage (last two samplings, Figures 3B,C). The ratio of C and N also varied greatly in the initial stage but finally declined with decomposition, and the significant differences among treatments also existed in the latal stage (Figure 3D).

**Table 1:** Results of the two-way ANOVA for the effect of treatment (T) and decomposition time (D*t*) on the variation of fine root decomposition traits of *Artemisia halodendron*.

**Parameter**	**Treatment (T)**		**Decomposition time (D** ***t*** **)**		**T** **×D** ***t***	
	**F**	**Sig**.	**F**	**Sig**.	**F**	**Sig**.
Mass remaining (%)	6.659	**0.002**	166.137	** <0.001**	1.553	0.151
C concentration (%)	1.948	0.149	16.515	** <0.001**	2.695	**0.011**
N concentration (%)	5.09	**0.008**	98.740	** <0.001**	3.680	**0.001**
C:N	3.948	**0.023**	41.892	** <0.001**	3.194	**0.003**
C remaining (%)	4.726	**0.017**	99.573	** <0.001**	2.631	**0.013**
N remaining (%)	8.709	** <0.000**	10.350	** <0.001**	1.455	0.186

*The bold values indicate significant differences of the fine root decomposition traits of A. halodendron under different treatments (ANOVA followed by LSD test, P < 0.05)*.

The mass remaining and N concentration were also affected by treatment (Table 1). The presence of mycelia stimulated fine root decomposition of *A. halodendron* in the late-stage except in the final sampling when the mass remaining did not differ significantly among treatments (Figure 3A). Treatment R + M and treatment M both increased the C concentration but decreased the N concentration in the last two samplings; thus, the C:N was promoted by treatment R + M and treatment M after 1 year of decomposition (Figures 3B–D).

Furthermore, there were significant interactions among treatment and decomposition times for C concentration, N concentration, and C:N (Table 1). This indicates that the presence of plant fine root or mycelia affected the dynamic during decomposition. For example, the C concentration varied among decomposition times for treatments R + M and M in the initial stage. In contrast, the C concentration increased gradually with decomposition in treatment S. In the following stage, the C concentration varied slightly for treatment S but gradually increased for treatments R + M and M (Figure 3B).

**Figure 4:**
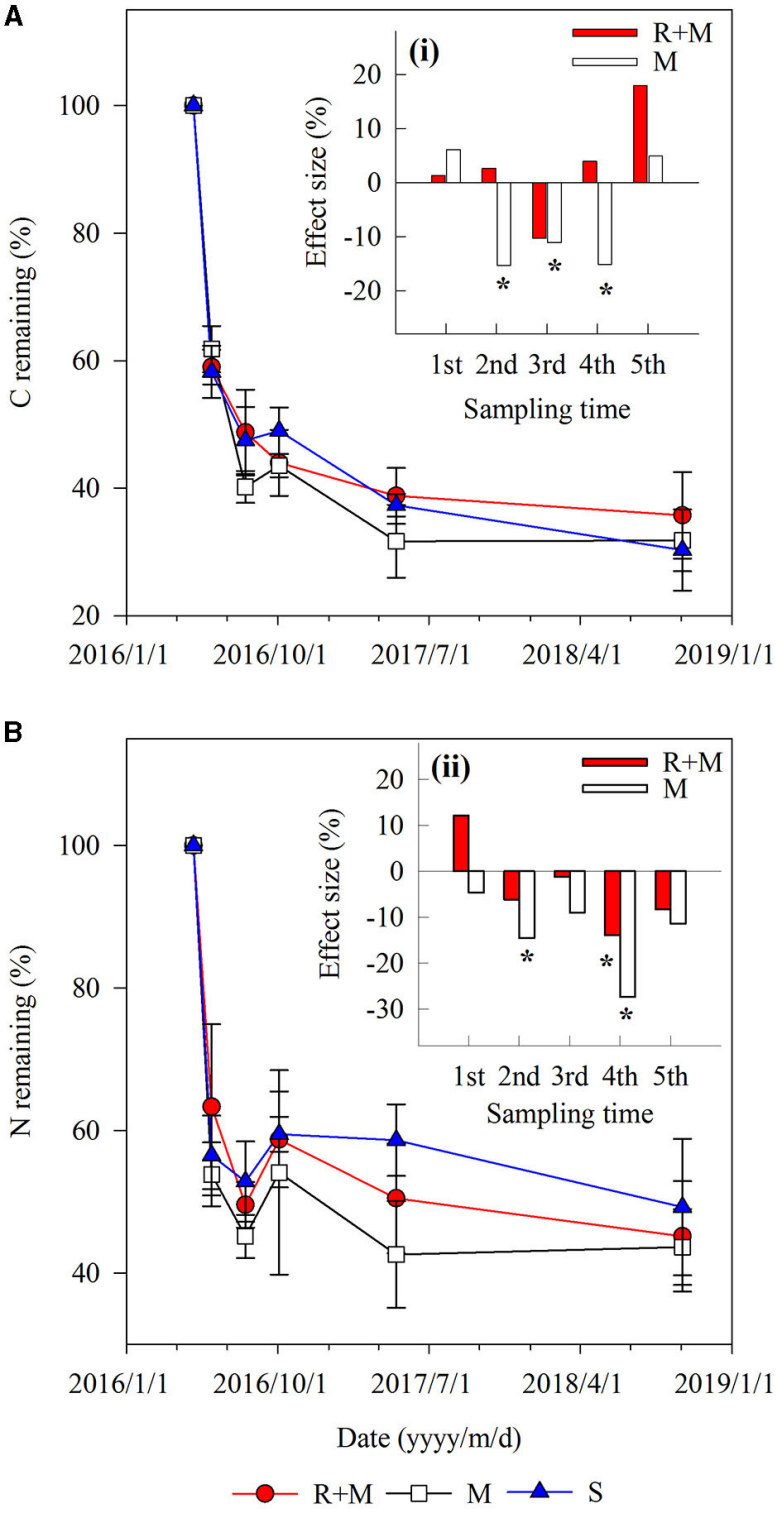
Carbon and nitrogen remaining during decomposition for fine roots of *A. halodendron* under the following three treatments: fine roots and mycelia (R + M), mycelia (M), and bulk soil (S). Values represent *M* ± *SD*. The small bar plot inset top-right indicates the effect size of R + M (red bar) and M (white bar) compared with S for C remaining **(A)** and N remaining **(B)**. Bars labeled with * indicate significant differences (*P* < 0.05) compared with the S treatment.

There were also interactions between treatment and decomposition time for the C remaining during decomposition (Table 1). Both the C remaining and the N remaining varied similarly with the mass remaining during decomposition. However, the dynamics of C remaining of the fine roots during decomposition differed among treatments. For example, the C remaining decreased gradually for treatment R + M at the end of the first growing season (from August 3 to October 3, 2016), whereas it slightly increased for treatment M and treatment S (Figure 4).

### Soil Properties Under the Litterbag

**Figure 5:**
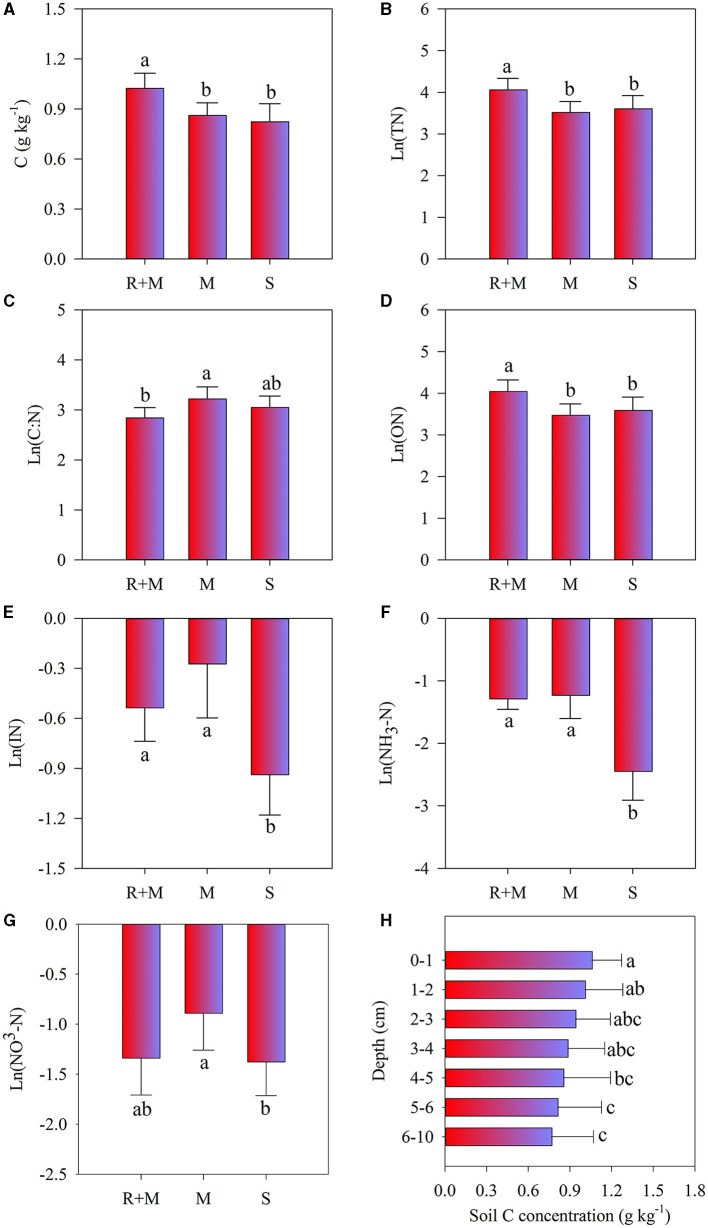
Soil properties of soil C concentration **(A)**, soil total-N (TN) concentration **(B)**, soil C:N ratio **(C)**, soil organic-N (ON) concentration **(D)**, soil inorganic-N (IN) concentration **(E)**, ammonium-N (NH4-N) concentration **(F)**, and nitrate-N (NO3-N) concentration **(G)** under the litterbag after 1 year of decomposition of the fine roots of *A. halodendron*. **(H)** indicates the concentration variation of soil C concentration among soil depths. Values represent *M* ± *SD*. Bars labeled with different letters differed significantly among the treatments (*P* < 0.05).

Two-way ANOVA showed that soil properties (except soil moisture) under the litterbag were all significantly affected by treatments, and only soil C concentration significantly differed with soil depth (Table 2). Moreover, no significant interactive effects among treatment and soil depth on soil properties were observed. The soil C, total-N, and organic-N under the litterbag were all significantly higher under treatment R + M than those under treatment M and under treatment S. The inorganic-N and ammonium-N under the litterbag were both significantly lower in treatment S than in the other treatments. The nitrate-N under the litterbag also varied significantly among treatments and was significantly higher under treatment R + M than that under treatment S. Soil C declined gradually but significantly with soil depth (Figure 5). However, this significant vertical pattern under the litterbag was only observed for C and not for other soil properties.

**Table 2:** Results of the two-way ANOVA for the effect of treatment (T) and soil depth (D) on the variation of soil moisture, soil carbon (C) concentration, soil total nitrogen (TN) concentration, soil C:N ratio, soil organic-N (ON) concentration, soil inorganic N (IN) concentration, ammonium N (NH_4_-N) concentration, and nitrate N (NO_3_-N) concentration.

**Parameter**	**Treatment (T)**		**Depth (D)**		**T × D**	
	**F**	**Sig**.	**F**	**Sig**.	**F**	**Sig**.
Moisture	0.790	0.457	0.077	0.998	0.126	1.000
C	8.538	**0.000**	3.205	**0.007**	0.854	0.595
ln(TN)	5.749	**0.005**	1.941	0.084	0.830	0.620
ln(C:N)	3.647	**0.030**	1.135	0.350	0.834	0.616
ln(ON)	5.967	**0.004**	1.903	0.090	0.878	0.572
ln(IN)	8.445	**0.000**	1.893	0.092	0.823	0.626
ln(NH_4_-N)	18.815	**0.000**	1.666	0.140	0.965	0.489
ln(NO_3_-N)	3.109	**0.050**	1.514	0.184	1.304	0.233

*Because the original soil moisture and C data were normally distributed, they were analyzed directly, whereas the other parameters were not distributed normally, and thus we analyzed these parameters after ln transformation*.

*The bold values indicate significant differences of soil properties under different treatments (ANOVA followed by LSD test, P < 0.05)*.

## Discussion

### Effect of Fine Roots and Mycelia on Fine Root Decomposition

#### Mass Loss

As a general pattern of litter decomposition, the decomposition rate is rapid in the early stage and then gradually slows (Camir et al., 1991; Olsson et al., 1996; Silver and Miya, 2001). The results of this study also fit this profile (Figure 3A). In this study, the mass loss of the fine roots of *A. halodendron* was as high as 41.7–45.3% at the initial stage of decomposition (33 days), which was higher than the previous study (Luo et al., 2016a). In addition, the mass loss after 1 year of decomposition in the current study ranged from 65.5 to 72%, which is also higher than the former studies (Luo et al., 2016a, 2020b). The relatively higher decomposition rate for the fine roots of *A. halodendron* would result from the patterns of precipitation over years (Luo et al., 2016a, 2020b).

The difference in mass loss between treatments in this study indicated that the presence of the mycelia significantly promoted the decomposition of the fine roots of *A. halodendron*. Existed study showed that, the presence of mycelia can retard the fine root decomposition *via* its changes on fine root quality (Langley and Hungate, 2003) or *via* the decrease in enzyme activities related to cellulose and lignin decomposition (Lin et al., 2019). Some studies demonstrated that the fine root decomposition would accelerate by the presence of mycelia (Pigott, 1982; Tu et al., 2006; Pritsch and Garbaye, 2011), which is consistent with the study. Research has shown that mycelia can promote the activity of soil microorganisms through the input of fresh C, thus promoting the decomposition of SOC, especially the inert C pool (Blagodatskaya and Kuzyakov, 2008; Kuzyakov, 2010; Zhang et al., 2018). It can be inferred that the excitation effect of mycelia may be an important reason for the accelerated root decomposition of *A. halodendron*.

However, the presence of mycelia in addition to the fine roots did not stimulate the root decomposition (Figure 3A). This may relate to other factors derived from the living fine roots. Root exudate is considered to have an important role in litter decomposition and the soil nutrient cycle (Nardi et al., 2002; Landi et al., 2006; Phillips et al., 2011; Yin et al., 2014), and the secondary metabolites of the exudates inhibit SOC decomposition by the suppression of soil microorganisms (Zhang et al., 2015; Zwetsloot et al., 2018). Thus, it can be inferred that that mycorrhizal and exudate effects on decomposition canceled each other out.

In addition, in arid and semi-arid regions with high water limitation, the water absorption by the root system during plant growth significantly reduces the soil moisture content (Schwinning and Ehleringer, 2001; Loik et al., 2004; Zhou et al., 2015). This reduction of soil moisture thereby decreased the root decomposition. In this study, soil moisture content after 1-year decomposition showed that, a relatively lower soil moisture content in treatment R+M (Supplementary Figure 1). Thus, it can be inferred that the combined effect of root exudates and water adsorption should reduce the promotion of root decomposition by mycelia, leading to the observation of no significant differences in the mass remaining between the R + M and S treatments (Figure 3A).

#### C and N Concentrations in Roots

In this study, root C concentration increased during decomposition (Figure 3B), this result was also consistent with the former study (Luo et al., 2016a). These dynamics may have occurred because of the rapid emission of nonstructural matter including phosphorus, potassium, and manganese at the early stage of decomposition (Lemma et al., 2007; Gómez-Muñoz et al., 2014). Contrastingly, nonstructural carbohydrates decomposed faster than structural carbohydrates such as lignin and cellulose (Camiré et al., 1991; Steinberger et al., 1995).

There was also a significant interaction between treatment and decomposition time for parameters of C concentration (Table 1). This indicates that the presence of fine roots and mycelia in the ingrowth core changed the variation of root C concentration during decomposition compared with treatment S (Figure 3B). Therefore, it can be hypothesized that the fine roots and mycelia influence the decomposition model of different components of litter, i.e., structural or non-structural carbohydrates. Determination of the stoichiometric characteristics of litter and identification of the microbial community during decomposition in future studies may provide a clearer explanation of this hypothesis.

The results of this study showed that the fine root N concentrations during decomposition in treatments R + M and M were both lower than those in treatment S (Figure 3C). This may be related to the promotion effect of mycelia on N mineralization (Phillips et al., 2011; Yin et al., 2014; Zhang et al., 2018, 2019). This explanation was supported by the soil properties under litterbags, which showed that the inorganic N in treatment R + M and treatment M were both higher than in treatment S (Figure 5E). In addition, it has also been demonstrated that mycorrhiza can absorb and utilize organic N from the refractory organic matter of plant litter *via* the associated saprophytic microorganisms (Colpaert and Van Laere, 1996). Therefore, the absorption of organic N by mycorrhiza from the decomposing root may be an important factor leading to the decreased N concentration in decomposing roots.

This study was conducted in semi-arid sandy grasslands with a typical water limitation; the root absorption of soil pore water can significantly reduce the soil moisture (Schwinning and Ehleringer, 2001; Loik et al., 2004; Zhou et al., 2015). The living roots would suppress the root N release during decomposition *via* this change in soil moisture, thereby leading to the observed difference of N remaining (N remaining in treatment S is higher than in treatment R + M and in treatment M) (Figure 4B).

### Effect of Fine Roots and Mycelia on Soil Properties Under the Litterbag

#### Soil C

There was a significant vertical pattern in soil C distribution under litterbags in which the SOC decreased gradually (Figure 5H). This result is consistent with the previous research (Luo et al., 2016a). The study of Liebmann et al. (2020) showed that the main form of C transformation from plant litter to the soil during decomposition was dissolved organic C rather than particulate organic C. The shift of plant C allocation to mycorrhizal fungi could promote carbon accumulation in soil, and the fine root litter could directly contribute to the process of stable soil organic matter formation (Langley et al., 2006). In this study, the experiment was conducted in the dune, the sandy soil texture allowed the quick leaching (Yao et al., 2013). Thus, the process of leaching the dissolved organic C from precipitation may be an explanation of this vertical pattern.

Meanwhile, the vertical pattern of soil C distribution was weakened in treatments R + M and M compared with treatment S (Supplementary Figure 2), which may be related to the living fine roots. It has been reported that plant roots increase the decomposition of soil organic matter but also promote the formation of stable soil organic matter in low-N ecosystems (Adamczyk et al., 2019). Studies have also shown that root exudates have a positive or negative priming effect on SOC (Kuzyakov, 2002); decomposition of SOC was promoted by the primary metabolites of root exudates (Rukshana et al., 2012; Girkina et al., 2018), which also can be inhibited by the secondary metabolites of root exudates (Zhang et al., 2015; Zwetsloot et al., 2018). The current study showed that there was no significant difference in soil C between treatment M and treatment S, but both were lower than that under treatment R + M (Figure 5A). This reveals that the existence of fine roots of *A. halodendron* has an accumulation effect on the soil C concentration. This accumulation only resulted from the fine root but not from the mycorrhiza. In addition, a previous study suggested that the mycelia could promote soil stable C accumulation due to its slower decomposition, which may become physically fractured but resist chemical decomposition, and then contribute directly to the formation of stable soil C (Langley et al., 2006). However, the presence of mycelia did not increase the soil C concentration, as the result of this study showed (Figure 5A). Therefore, it can be inferred that exudates from the fine roots of *A. halodendron* are one of the important factors in soil carbon accumulation in Horqin sandy land. However, this inference needs to be specifically tested in subsequent studies.

In addition, there was no significant difference in soil C concentration among treatments M and S (Figure 5A). Previous studies have shown that the fresh C released by mycorrhiza can promote the decomposition of soil C, especially the inert C (Blagodatskaya and Kuzyakov, 2008; Kuzyakov, 2010; Zhang et al., 2018). Meanwhile, the rapid turnover of the mycorrhiza stimulates the accumulation of soil C (Heinemeyer et al., 2007; Pickles et al., 2010; Cairney, 2012). Therefore, another tradeoff between promotion on SOC decomposition and stimulation of SOC accumulation may explain the lack of significant difference in soil C concentrations among the M and S treatments (Figure 5A).

#### Soil N

There was no vertical effect on soil N content under the litterbag (Table 2), which indicates that the N distribution under the litterbag was not affected by the fine root decomposition. This finding is consistent with the previous research (Luo et al., 2016a), which showed that root decomposition of *A. halodendron* for both the fine roots (≤2 mm) and the coarse roots (>2 mm) did not affect soil N concentration under litterbags. This result may be related to leaching interaction with resorption by plant roots (Luo et al., 2016a). In this study, there was no significant difference in the soil total N and the organic N between M and S treatments (Figures 5B,D). This indicates that the existence of the mycelia did not affect soil total N and organic N concentrations. However, these concentrations were both enhanced by the treatment of R + M (Figures 5B,D). Therefore, it can be hypothesized that the organic N in the soil is derived from the living fine roots. Moreover, the living roots can stimulate the activities of microorganisms in rhizosphere soil by the release of root exudates (Landi et al., 2006; Phillips et al., 2011; Yin et al., 2014). The turnover of the enhanced microbes may be a possible reason for the organic N accumulation. In addition, the fast turnover rate of the fine roots of *A. halodendron* (Huang et al., 2012; Luo et al., 2016b) would contribute N to the soil.

The study of Zhang et al. (2019) demonstrated that the soil inorganic N pool was significantly higher in soil with fine roots plus mycelia in the alpine forest than in bulk soil in the eastern Tibetan Plateau of China. This supports the findings of our study in degraded grassland (Figure 5E). The explanation for this difference was that the release of new C from the fine root or the mycelia stimulated the N cycle (Phillips et al., 2011; Yin et al., 2014; Zhang et al., 2018). For example, the root exudate from *Cupressus funebris* significantly enhanced the concentration of alkali-hydrolysable N of potted *Toona sinensis* (Yi et al., 2019). An *in situ* observation study of mature loblolly pine (*Pinus taeda*) found that the enhanced root exudation stimulated by the carbon dioxide (CO_2_) enrichment can accelerate the turnover of N pools in the rhizosphere (Phillips et al., 2011).

## Conclusions

During the fine root decomposition of *A. halodendron* in a degraded sandy grassland, the loss of the mass and the release of litter C and N were all stimulated by the presence of mycelia. Under the litterbag, the mycelia significantly stimulated soil inorganic N (ammonium-N and nitrate-N) accumulation but the presence of fine roots weakened the accumulation of soil nitrate-N. The presence of living roots and associated mycelia strongly affected the process of root decomposition and matter release in the litter-soil system.

## Data Availability Statement

The original contributions presented in the study are included in the article/Supplementary Material, further inquiries can be directed to the corresponding authors.

## Author Contributions

YL and ZD designed the study. XL, LC, and HH conducted the field trial. LC and HH performed the laboratory analysis. XL, YL, and ZD were responsible for the statistical analyses. XL and YL wrote the original draft manuscript. XL, YL, YW, and ZD critically reviewed and edited the preliminary draft manuscript. Finally, all the authors approved the final version of the manuscript.

## Conflict of Interest

The authors declare that the research was conducted in the absence of any commercial or financial relationships that could be construed as a potential conflict of interest.

## Publisher's Note

All claims expressed in this article are solely those of the authors and do not necessarily represent those of their affiliated organizations, or those of the publisher, the editors and the reviewers. Any product that may be evaluated in this article, or claim that may be made by its manufacturer, is not guaranteed or endorsed by the publisher.

## Supplementary Material

Supplementary Figure 1 Vertical patterns of soil moisture content under litterbags after 1 year of decomposition of the fine roots of *A. halodendron* of the following three treatments: fine root and mycelia (R + M), mycelia (M) and bulk soil (S). Values represent *M* ± *SD*.

Supplementary Figure 2 Vertical patterns of soil C concentration under litterbags after 1 year of decomposition of the fine roots of *A. halodendron*. Values represent *M* ± *SD*. Bars labeled with different letters differed significantly among treatments (*P* < 0.05). Soil C concentration decreased gradually with the increase of soil depth. This vertical pattern was significant for the bulk soil (S) and was weakened by the presence of fine root + mycelia (R + M) and of mycelia (M) under the *A. halodendron* canopy in Horqin sandy land, northeast China.
