# Supplementary material for: Effect of Root and Mycelia on Fine Root Decomposition and Release of Carbon and Nitrogen Under Artemisia halodendron in a Semi-arid Sandy Grassland in China
Source: Front Plant Sci. 2021 Sep 1;12:698054. doi: 10.3389/fpls.2021.698054 (PMC8442746; doi:10.3389/fpls.2021.698054)
Supplement: Supplementary Figure 1 — Vertical patterns of soil moisture content under litterbags after 1 year of decomposition of the fine roots of A. halodendron of the following three treatments: fine root and mycelia (R + M), mycelia (M) and bulk soil (S). Values represent M ± SD. [file Data_Sheet_1.pdf]

Supplementary Material

Effect of root and mycelia on fine root decomposition and release of carbon and nitrogen under Artemisia halodendron in a semi-arid sandy grassland in China

Xinping Liu[1], Yongqing Luo[1*], Li Cheng[1], Hongjiao Hu[1], Youhan Wang[2] and Zhong Du[2,3*]

[1]Naiman Desertification Research Station, Northwest Institute of Eco-Environment and Resources, Chinese Academy of Sciences, Lanzhou, China,

[2] School of Geographical Sciences, China West Normal University, Nanchong, China

[3]Forest Dynamics, Swiss Federal Institute for Forest, Snow and Landscape Research WSL, Birmensdorf, Switzerland

*Corresponding Author:

Phone: +86 817 2568683

Fax: +86 817 2568683

Email: duzhong@cib.ac.cn

Phone: +86 931 4967217

Fax: +86 931 4967219

Yongqing Luo, luoyongqing@nieer.ac.cn

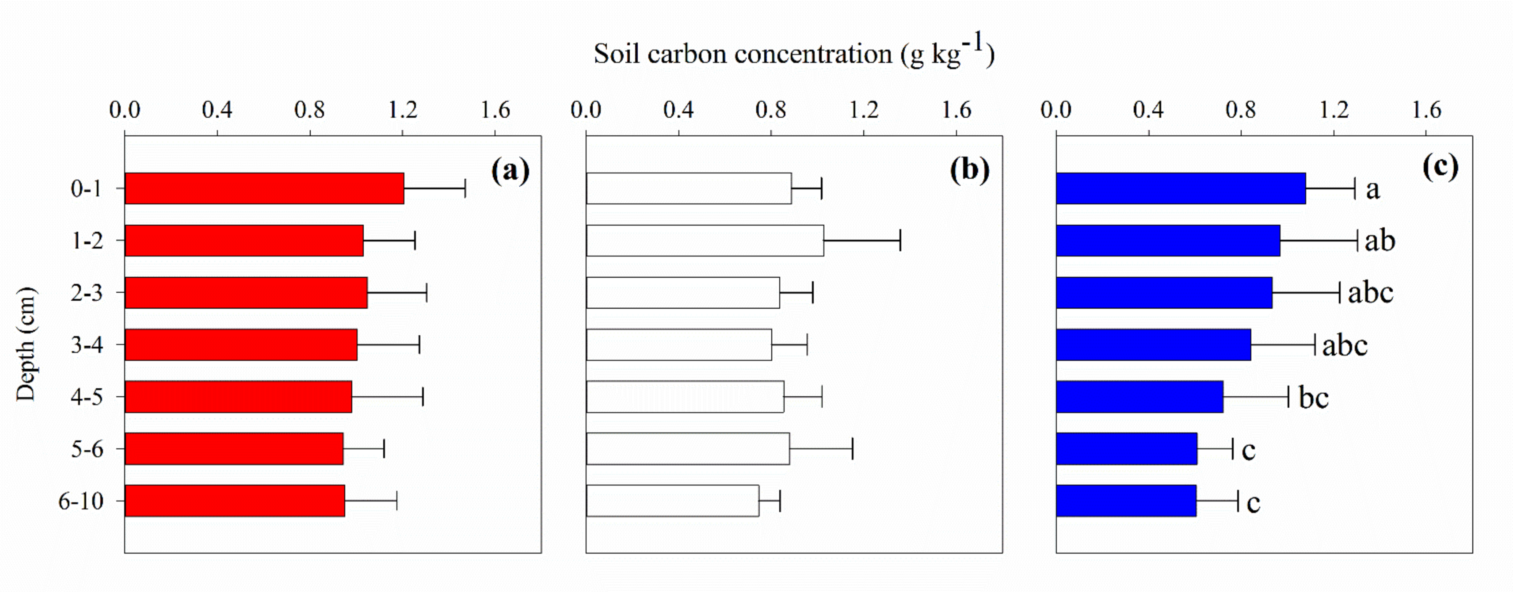

Supplementary Fig. 1 Vertical patterns of soil moisture content under litterbags after 1 year of decomposition of the fine roots of *A. halodendron* of the following three treatments: fine root and mycelia (R+M), mycelia (M) and bulk soil (S). Values represent $M \pm SD$.

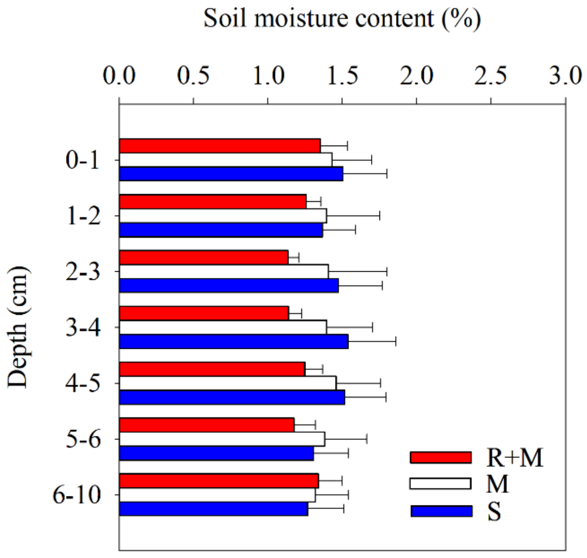

Supplementary Fig. 2 Vertical patterns of soil C concentration under litterbags after 1 year of decomposition of the fine roots of *A. halodendron*. Values represent *M* ± *SD*. Bars labeled with different letters differed significantly among treatments ($P < 0.05$). Soil C concentration decreased gradually with the increase of soil depth. This vertical pattern was significant for the bulk soil (S) and was weakened by the presence of fine root + mycelia (R+M) and of mycelia (M) under the *A. halodendron* canopy in Horqin sandy land, northeast China.
